# Report of a Case of Pneumatic Aspiration of Pericardial Sac, in Cook Co. Hospital

**Published:** 1874-11-01

**Authors:** D. A. K. Steele

**Affiliations:** Resident Physician


### REPORT OF A CASE OF PNEUMATIC ASPIRATION OF PERICARDIAL SAC, IN COOK COUNTY HOSPITAL.

By D. A. K. Steele, Resident Physician.

GENTLEMEN: The case I will call your attention to is one adverted to by Prof. Johnson, two weeks ago, before your Society, in connection with his report on pneumatic aspiration.

The patient, Edward S., age forty-five; laborer; native of Germany; was admitted to the hospital September 22, 1874, in the evening. Stated that he enjoyed good health until ten weeks prior to admission, when while employed in a lager beer saloon, and sweating profusely, he went into the ice cellar to cool off, and took a violent cold; had several slight chills, followed by pretty high fever, when he was attacked by severe cramping pains in back and left side in region of heart; pain was aggravated by a deep inspiration; had no cough; appetite became impaired, bowels costive; breathing labored; pain in left chest and difficulty of respiration continued for about four weeks, when he began to convalesce; commenced to work as a cook, again exposing himself to sudden changes of temperature; sleeping in a damp basement. After being so employed for about three weeks he was attacked with pain in left chest, similar in character to first attack; began to cough, expectorating a white frothy sputum. For past two weeks breath has been gradually becoming shorter and more labored; rests poorly at night; appetite poor; bowels costive. Has been a moderate drinker for a number of years; family history devoid of tuberculous taint; never had syphilis, and gives no history of rheumatism.

On admission.—Patient, a large, well nourished man; lies on left side with shoulders elevated and thighs flexed on abdomen; breathing hurried and laborious; face anxious and cyanosed; skin cool and moist; tongue flabby, tremulous and covered with a brownish coating; pulse 132, small, thready and irregular; respiration 28 per minute; temperature a little below normal.

On physical examination.—Inspection reveals bulging of left chest and præcordial region; epigastric protrusion; partial obliteration of left intercostal spaces and decided loss of motion in left chest; heart communicates no thoracic shock; palpation gives an absence of vocal fremitus in left chest anteriorly, while posteriorly the fremitus is exaggerated.

Percussion reveals complete dullness in left chest anteriorly over a somewhat irregular triangle, the base of which would correspond to a transverse line drawn from left axillary region three inches below left nipple to a point two inches to right of tiphoid appendix. Apex at a point about five inches above and one and one-half inches to right of left nipple. The dullness was but slightly altered by postural change; behind get fair resonance; in right chest resonance slightly increased.

On auscultation.—Broncho-vesicular respiration throughout right chest; at apex of left lung anteriorly get bronchial breathing; below third rib an absence of all lung sounds; behind over upper one-third of chest bronchial respiration; over middle broncho-vesicular with mucous rales, and over base bronchial again with occasional fine creptitant rales, and occasional friction sounds.

On auscultating cardic region find an absence of all heart sounds; along the right border of sternum get a few to and fro pericardial friction sounds. Summing up the physical signs we readily arrived at a diagnosis of acute pericarditis with a large serous effusion. Applied warm jacket poultice and gave alcoholic stimuli. Next morning patient not being relieved and dyspnœa increasing dispatched a messenger for Dr. Johnson, thinking that an attempt should be made to relieve the imminent danger of death from dyspnœa by aspirating the pericardial sac and removing a portion of the fluid compressing the lungs. Prof. Johnson arrived in the evening (patient in the meantime having diffusible stimuli, carb. ammonia, quinia and camphor), and confirmed diagnosis, and as the danger of sudden death was imminent determined on tapping the pericardium with the aspirator as a means of temporary relief. Patient was placed in a sitting posture, and a fine canula connected with a vacuum, was introduced in fifth intercostal space two inches to left of sternum, and carefully carried inwards, upwards and backwards, until it had penetrated the thoracic wall about two inches, when a few drops of bloody serum escaped; about one ounce was removed when the canula was withdrawn. The immediate effect of the aspiration was to quicken and strengthen the circulation as determined by the radial pulse. Heart continues intermittent. Supposition is that the canula entered a little pouch or pocket formed at base of pericardium by plastic effusion and did not enter the main collection of fluid. Patient was continued on stimulants, chest painted with tincture iodine; was also given saline cathartics and diuretics.

24th. Expresses himself as feeling a little better; says he can breathe easier; continued treatment with but little change of symptoms or physical signs until the 28th, when a pleuro-pneumonia of left chest manifested itself, with a corresponding increase of the gravity of the symptoms. A warm jacket poultice was kept continuously applied, and free stimulation resorted to with the application of artificial warmth to extremities. On the 30th a consultation of the attending physicians was held, and it was determined to again resort to aspiration as a dernier ressort. Prof. Johnson introduced a fine canula near point of previous puncture, carrying the point in same direction, but a little higher than before, until the point had passed into chest about two inches, when on turning the stopcock the skillful operator was rewarded by seeing a full stream of bloody serum flowing into the receiving bottle, from pericardial sac. Eleven and one-half ounces were withdrawn, when the canula was removed, no unpleasant symptoms occurring. The immediate effect of the operation was to relieve the turgescence of the superficial vessels and to steady and strengthen the heart's action. Breathing became easier and the apex beat of the heart could be seen for the first time striking a little to the left and higher than normal; cardic sounds distinctly heard; bulging of intercostal spaces not so marked. Half an hour after the operation pulse 132; respiration 28 per minute.

October 1st. Pneumonic inflammation more extensive; extremities cold; radial pulse imperceptible; heart's action feeble, irritable, and irregular; general cyanosis; cannot retain anything on his stomach; is delirious; gradually sank and died at 1 A. M. following morning.

Autopsy. Fourteen hours after death entire body cyanotic; antero-mesial incision, superficial structures normal in appearance. On lifting up sternum found pericardial sac immensely distended with fluid, and occupying nearly the whole anterior portions of left chest and overlapping the right lung for two or three inches; diaphragm depressed; moderate pleuritic effusion in both chests. On opening pericardium found it much thickened and congested, containing a considerable amount of bloody serum; point of last puncture readily seen; point of previous puncture closed by adhesive inflammation; heart hypertrophied, dilated, apparently inflamed; right side distended with venous coagula, entire surface covered with tenacious coagulated lymph arranged in segregated layers resembling villi, varying in depth from one-half to one inch and adhering so firmly to walls of heart as to be with difficulty removed; pericardium adherent to heart around base by means of this plastic deposit. Left lung throughout pneumonic, in a state of red hepatization, quite friable and firmly adherent to chest walls by recent pleuritic adhesions; right lung congested; kidneys, liver and spleen very much congested; other organs not examined.

In answer to questions from members Dr. Steele stated that no heart sounds were heard until after the second tapping, but that the valves were, probably, not diseased. The heart itself was not weighed, but the heart and sac together weighed four pounds.

The patient, when in health, weighed one hundred and seventy pounds, and was five feet eleven inches high. Before operating, the patient was advised fully as to the operation, and that it was only expected to give temporary relief,—that in itself the operation was not curative. A needle, a little larger than the one used in the hypodermic syringe, was employed.

In reply to an inquiry by Dr. Merriman, it was stated that the patient had never had rheumatism, but had been a large healthy man until three months ago. Dr. Hamill expressed some doubts as to the advantage of aspiration, or its expediency. If there is absorption or removal of the fluid, may there not then be such adhesions as to hasten death?

Dr. F. H. Davis asked whether there was any effusion into the pleural cavities, and Dr. Steele said that there were twenty-five to thirty ounces in each cavity, the latter amount in the right chest.

Dr. F. H. Davis.—It seems as though one chief danger in this operation would arise from the liability of the puncture to allow a dribbling of the fluid into the pleural sac and thus excite a pleural inflammation, but of course the character of the fluid effused would influence to some extent this result. If a purulent fluid, the danger of a bad result would be much greater.

One of the members called attention to the fact that this patient, from the history given us, seems to have had a *primary* and *idiopathic pericarditis*, which is certainly very rare. In the morbid specimen, he saw no deposits of lymph upon the outer surface of the pericardial sac; the cardiac portion of the sac was everywhere thickly covered by the exudation.

Dr. Hyde.—So far as known this operation is the first one of the kind ever done in Chicago, and must have required the courage if not the audacity which that great physician Trousseau exhibited, who boldly plunged the needle into the pericardial fluid long before Dieulafoy had established the present method and processes.

A discussion then ensued between Drs. Simon, Merriman and Hyde as to the probable reason why the fluid that had been drawn off was saneous.

